# A Synthetic Cyclized Antimicrobial Peptide with Potent Effects against Drug-Resistant Skin Pathogens

**DOI:** 10.1021/acsinfecdis.2c00598

**Published:** 2023-05-03

**Authors:** John Kerr White, Soumitra Mohanty, Taj Muhammad, Magdalena de Arriba Sanchez de la Campa, Wael E. Houssen, Natalia Ferraz, Ulf Göransson, Annelie Brauner

**Affiliations:** † Department of Microbiology, Tumor and Cell Biology, Karolinska Institutet, SE-171 77 Stockholm, Sweden; ‡ Division of Clinical Microbiology, Karolinska University Hospital, SE-171 76 Stockholm, Sweden; § Pharmacognosy, Department of Pharmaceutical Biosciences, Uppsala University, Biomedical Centre, Box 591 SE-75124 Uppsala, Sweden; ∥ Institute of Medical Sciences, University of Aberdeen, Ashgrove Road West, Foresterhill, Aberdeen AB25 2ZD, U.K.; ⊥ Department of Chemistry, University of Aberdeen, Meston Walk, Aberdeen AB24 3UE, U.K.; # Nanotechnology and Functional Materials, Department of Materials Science and Engineering, Uppsala University, Box 35, SE-75103 Uppsala, Sweden

**Keywords:** keratinocytes, MRSA, synthetic antimicrobial peptide, LL-37, bactericidal peptide, wound closure

## Abstract

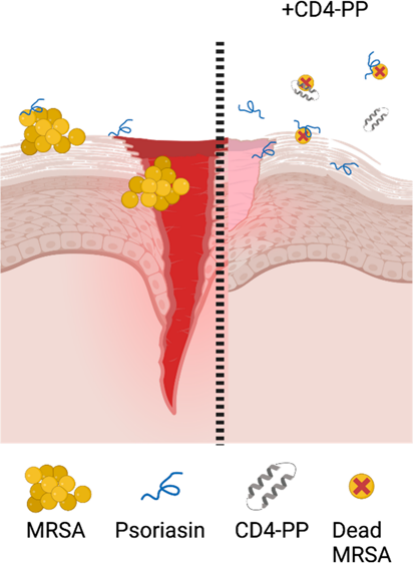

Dermal infections requiring treatment are usually treated with conventional antibiotics, but the rise of bacterial resistance to first-line antibiotics warrants alternative therapeutics. Here, we report that a backbone-cyclized antimicrobial peptide, CD4-PP, designed from the human host defense peptide LL-37, has strong direct antibacterial effects on antibiotic sensitive as well as resistant-type strains and clinical isolates of common skin pathogens in the low (<2) μM range. In addition, it influences innate immunity in keratinocytes, and treatment with CD4-PP is able to clear bacterial infections in infected keratinocytes. Additionally, CD4-PP treatment significantly reduces the wound area in a lawn of keratinocytes infected with MRSA. In conclusion, CD4-PP has the potential to serve as a future drug treating wounds infected with antibiotic-resistant bacteria.

The skin is the body’s primary barrier against pathogens, but its protective ability may be compromised by wounds. These can easily become infected, mainly by *Staphylococcus aureus* and increasingly often with methicillin-resistant *S. aureus* (MRSA) strains. Although infection does not always require treatment, widespread antibiotic resistance demands alternative therapeutics. Antimicrobial peptides (AMPs) could be one such alternative.^1^ They possess potent bactericidal properties on a broad range of human pathogens,^2^ and some AMPs have potent immune-regulatory impact. Despite their broad range of effect, resistance is seldom observed.^3^

We recently demonstrated that the novel cyclized AMP, CD4-PP, is a potent host immunomodulator having strong antibacterial activity in the context of urinary tract infection.^4^ Considering the high incidence of dermal infections,^5^ we wished to establish the possible use of CD4-PP for dermatological application. We evaluated the direct effect of CD4-PP against common skin pathogens, including those that are drug-resistant. In addition, the immunomodulatory and wound healing effects during infection and noninfected state were evaluated *in vitro* with human keratinocytes.

We here demonstrate that CD4-PP is active against common skin pathogens with MICs of 0.78–1.56 μM (Table 1) for both type strains and clinical isolates (*n* = 1 and *n* = 20, respectively) in concentrations far below cellular toxicity (25 μM).

**Table 1:** Minimum Inhibitory Concentrations (MICs) of CD4-PP toward Drug Sensitive and Resistant Skin Pathogens

species	type strain	MIC (μM)	#clinical isolates with same or lower MIC (%)
Group A *Streptococci*	ATCC 19615	0.78	20/20 (100%)
*Staphylococcus aureus*	ATCC 29213	0.78	18/20 (90%)
Methicillin-resistant *S. aureus*	CCUG 31966	1.56	20/20 (100%)
MDR *Acinetobacter baumannii*	N/A	1.56	20/20 (100%)

The efficacy of CD4-PP against group A streptococci (GAS), antibiotic-sensitive and methicillin-resistant *S. aureus*, as well as multidrug-resistant (MDR) *Acinetobacter baumannii* clinical strains is noteworthy as the development of bacterial resistance to AMPs is rare.^3,6^ In addition, when challenging CD4-PP-sensitive *S. aureus*, MRSA, and GAS for 1 week, no change in MIC was observed, thereby indicating that resistance to CD4-PP is not quickly established. This indicates that CD4-PP is effective against antibiotic-resistant bacterial strains, which otherwise are difficult to treat.

Many AMPs are known to act directly on the bacterial membrane and cause cell lysis.^6^ To visualize the effect of CD4-PP on the morphology of *S. aureus*, GAS, and MRSA-type strains, scanning electron microscopy (SEM) was used. The peptide induced the formation of blebs and ruffling of the bacterial surface compared with controls (Figure 1A–F). The blebbing pattern was comparable with what we had previously observed for *E. coli*.^4^ This suggests that CD4-PP has similar bacterial membrane deformation potential against both Gram-negative and Gram-positive bacteria.

**Figure 1:**
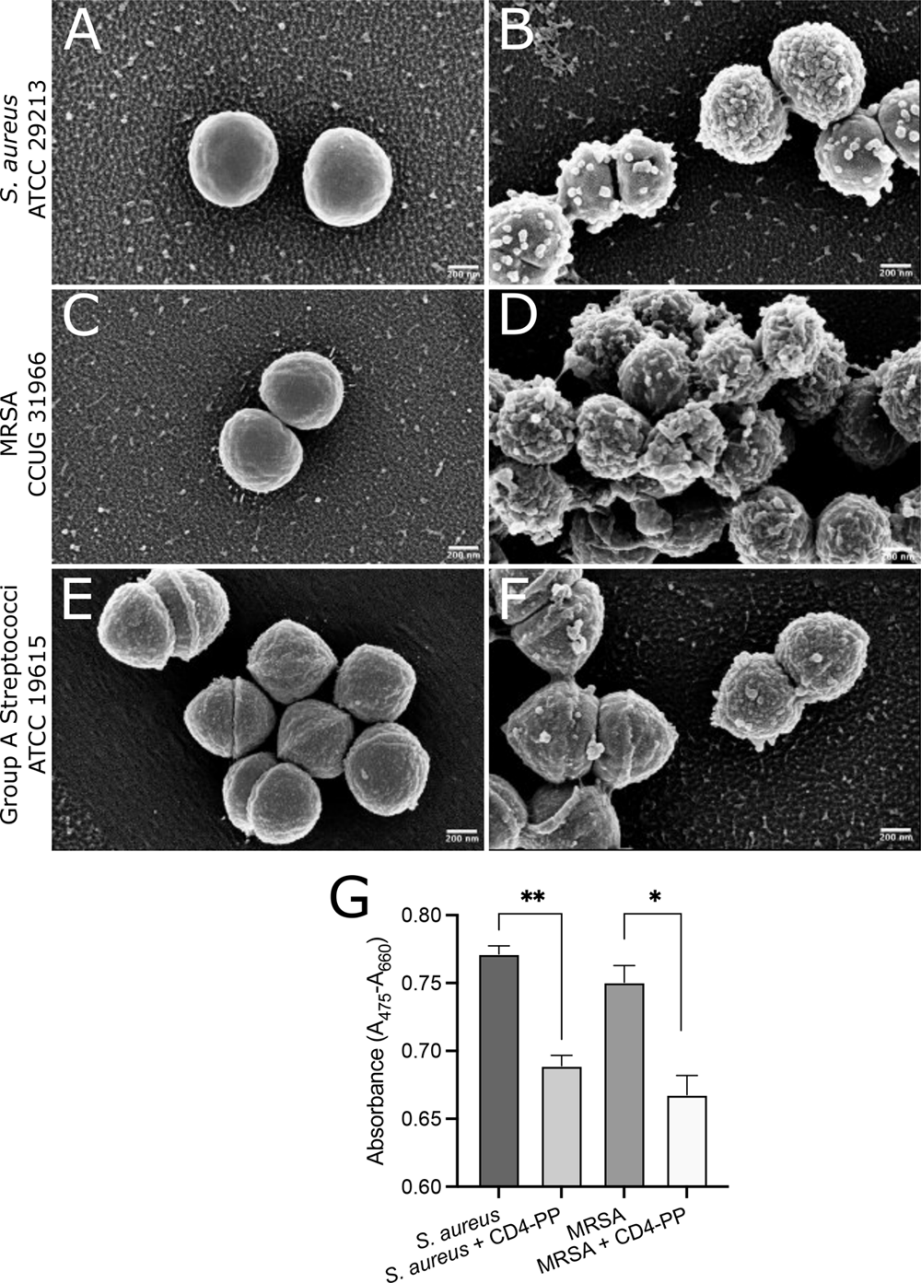
CD4-PP induces membrane damage and decreases bacterial metabolic activity. Representative scanning electron microscopy images of (A,B) *S. aureus* ATCC 29213, (C,D) MRSA CCUG 31966, and (E,F) group A streptococci (GAS) ATCC 19615 with or without CD4-PP treatment. Blebs are seen on the surface of all CD4-PP-treated bacteria compared with controls. (G) Metabolic activity expressed as conversion of tetrazolium salt XTT to a colored formazan derivative by *S. aureus* (*n* = 3) and MRSA (*n* = 3) with or without CD4-PP treatment (**p < 0.01, *p < 0.05, unpaired *t* test).

In addition to their direct bactericidal action, AMPs may also target metabolic activities.^7^ To investigate whether CD4-PP induced alterations to bacterial metabolism, the metabolic activity of *S. aureus* and MRSA-type strains exposed to CD4-PP was assessed. We observed that the addition of CD4-PP significantly reduced bacterial metabolic activity in both *S. aureus* and MRSA (Figure 1G). The combined effect of CD4-PP on bacterial membranes and metabolism indicates that it can slow or even inhibit bacterial growth.^8^ However, this might act as a double-edged sword as it is known that bacteria with decreased metabolic activity are less susceptible to certain antibiotics.^9^

Infection provokes an immune response by keratinocytes via the release of endogenous AMPs to thwart bacterial invasion.^10^ However, AMP expression and release can also be induced by other cationic compounds, which might act to prime the immune system before bacterial attack.^11^ Considering that CD4-PP is a synthetic cationic AMP derived from endogenous LL-37, we investigated whether CD4-PP could induce endogenous AMP production. Keratinocytes, HaCaT cells, infected with *S. aureus* and treated with CD4-PP showed an increase in the expression of *S100A7*, encoding for endogenous psoriasin (Figure 2A), while this was not the case in MRSA- or GAS-infected cells (Figure 2B,C). Previous studies have shown that psoriasin is preferentially active against certain bacterial species compared with other AMPs.^12^ Therefore, the differences observed in psoriasin expression in HaCaT cells might be due to specificity of species and strains. When stimulating uninfected cells, CD4-PP did not induce *S100A7* expression (Figure 2D). Increased *S100A7* in uninfected keratinocytes is not a detriment, as excess psoriasin peptide in uninfected skin is associated with other chronic inflammatory skin disorders, including psoriasis.^13^ Although MRSA infection did not induce increased *S100A7* on the gene level, we observed a significant increase on the protein level (Figures 2E,F), which could be due to differential regulation in the gene expression and the time point used in the analysis. In addition, we observed a colocalization of psoriasin released from keratinocytes with bacteria during infection, thus further supporting that the released psoriasin was killing the bacteria (Supplementary Figure 1A).

**Figure 2:**
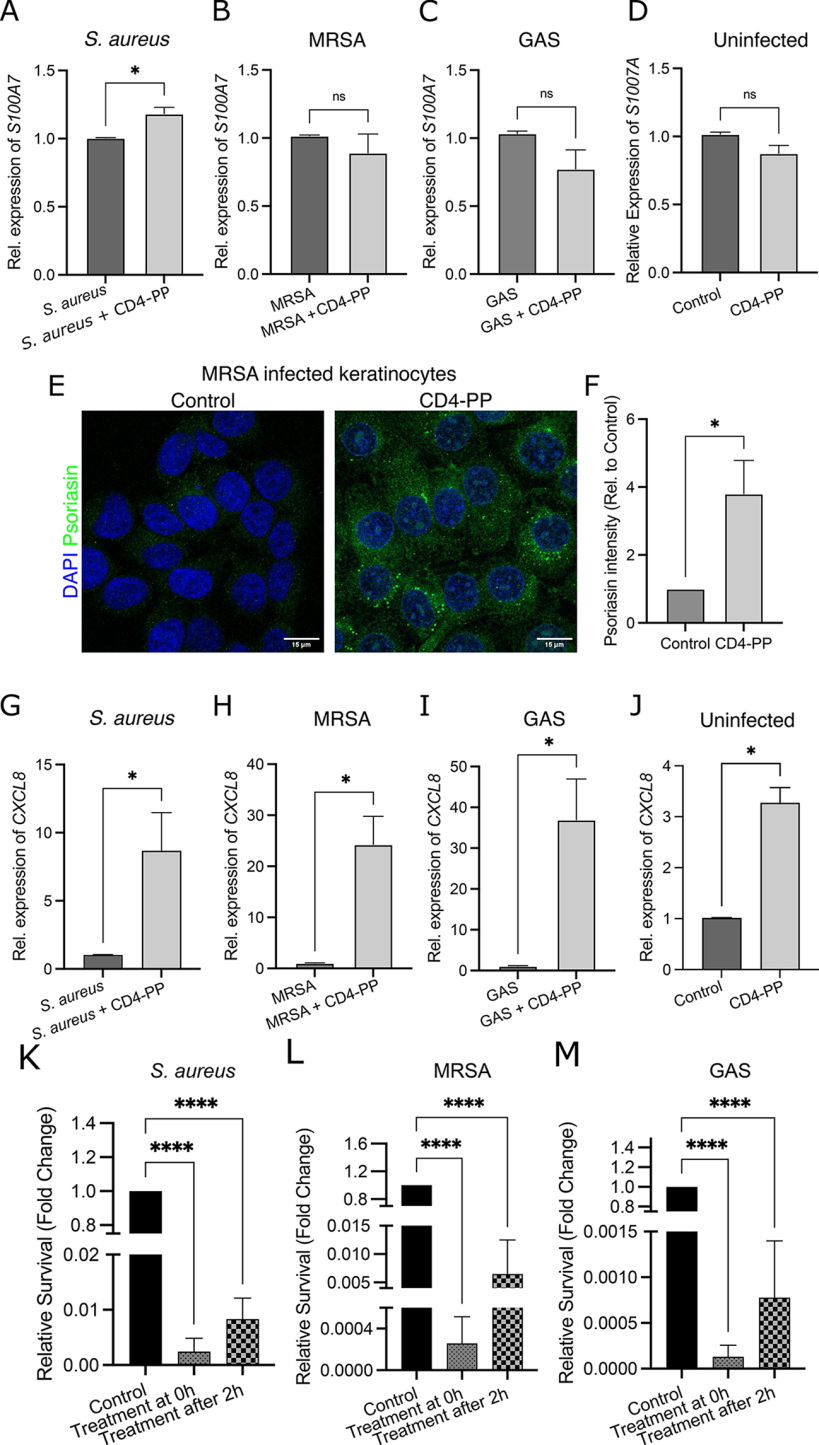
Increased psoriasin and *CXCL8* expression in keratinocytes stimulated with CD4-PP contributes to a decrease in infection. (A) During *S. aureus* infection, keratinocytes showed an increase in *S100A7* levels when treated with CD4-PP (*p < 0.05, paired *t* test). No differences in *S100A7* expression were observed in (B) MRSA- or (C) GAS-infected keratinocytes nor in (D) uninfected keratinocytes treated with CD4-PP. (E) Representative images of keratinocytes infected with MRSA with or without CD4-PP treatment depicting psoriasin peptide (green) and nucleus (blue). (F) Densitometric analysis of psoriasin staining in MRSA-infected samples showed a significant increase of keratinocytes treated with CD4-PP (*p < 0.05, unpaired *t* test). CD4-PP increased the expression of *CXCL8* in keratinocytes infected with (G) *S. aureus* (ATCC 29213), (H) MRSA (CCUG 31966), and (I) GAS (ATCC 19615) and (J) uninfected keratinocytes (*p < 0.05, paired *t* test). Survival of skin pathogens is shown for (K) *S. aureus* (ATCC 29213), (L) MRSA (CCUG 31966), and (M) GAS (ATCC 19615) after infecting keratinocytes. The survival in treatment groups is relative to untreated control. CD4-PP was initiated at the same time as infection (treatment at 0 h) or after 2 h of infection (treatment after 2 h) (****p < 0.0001, one-way ANOVA). Relative mRNA expression of target genes was performed in at least three independent sets in duplicate or triplicate. Microscopy imaging and densitometry analysis were performed in three independent experiments, with each experiment consisting of 4–5 random view fields. The average integrated density of each cell per set used for statistical analyses.

In addition to AMPs, stressed keratinocytes release chemokines and cytokines to recruit immune cells, such as resident neutrophils, to the site of infection.^3^ Stimulation of infected keratinocytes with CD4-PP revealed a significant increase in the expression of the neutrophil recruiter *CXCL8*, encoding for IL8 (Figures 2G,I). Interestingly, stimulation of uninfected keratinocytes with CD4-PP also revealed a significant increase in *CXCL8* expression (Figure 2J). The increase in *CXCL8* expression suggests that there will be an influx of neutrophils to the treatment site, thereby leading to an enhanced immune response.

During most dermal infections, bacteria bind to the surface of the epithelium.^14^ Therefore, we investigated the possible clearance of the aforementioned skin pathogens from keratinocytes during infection. To mimic a natural situation, we used two time points: treatment with CD4-PP at the start of infection or two hours postinfection. In addition, we utilized two cell types which are present in wounds: keratinocytes and resident macrophages. Human keratinocytes (HaCaT) and differentiated monocytes (dTHP1s), regarded as macrophages, were infected with the type strains of *S. aureus*, MRSA, or GAS. Interestingly, irrespective of when treatment was given to keratinocytes or macrophages, a clear decrease in bacterial survival was observed for *S. aureus*, MRSA, and GAS (Figure 2K,M and Supplementary Figure 1B–D).

To investigate the possible ability of CD4-PP to stimulate keratinocyte proliferation to aid in closing an open wound, we assessed whether CD4-PP would influence cell proliferation. Unexpectedly, we observed a significant decrease in the proliferation marker Ki-67 in CD4-PP-treated keratinocytes compared with untreated cells on both the mRNA and protein level (Figures 3A–C). Ki-67 has multiple roles depending on its localization and cell cycle state. Increased levels of Ki-67 are associated with cell proliferation as it becomes localized to the nucleus during cell division where it organizes heterochromatin.^15^ In contrast, a decrease in Ki-67, such as the one observed in this study, is associated with a reduction in cell proliferation and slower cell division.^16^ However, it should be noted that Ki-67 expression has mostly been studied in regard to cancer,^17^ and cells depleted of Ki-67 are still able to proliferate, albeit at a slower rate.^18^

**Figure 3:**
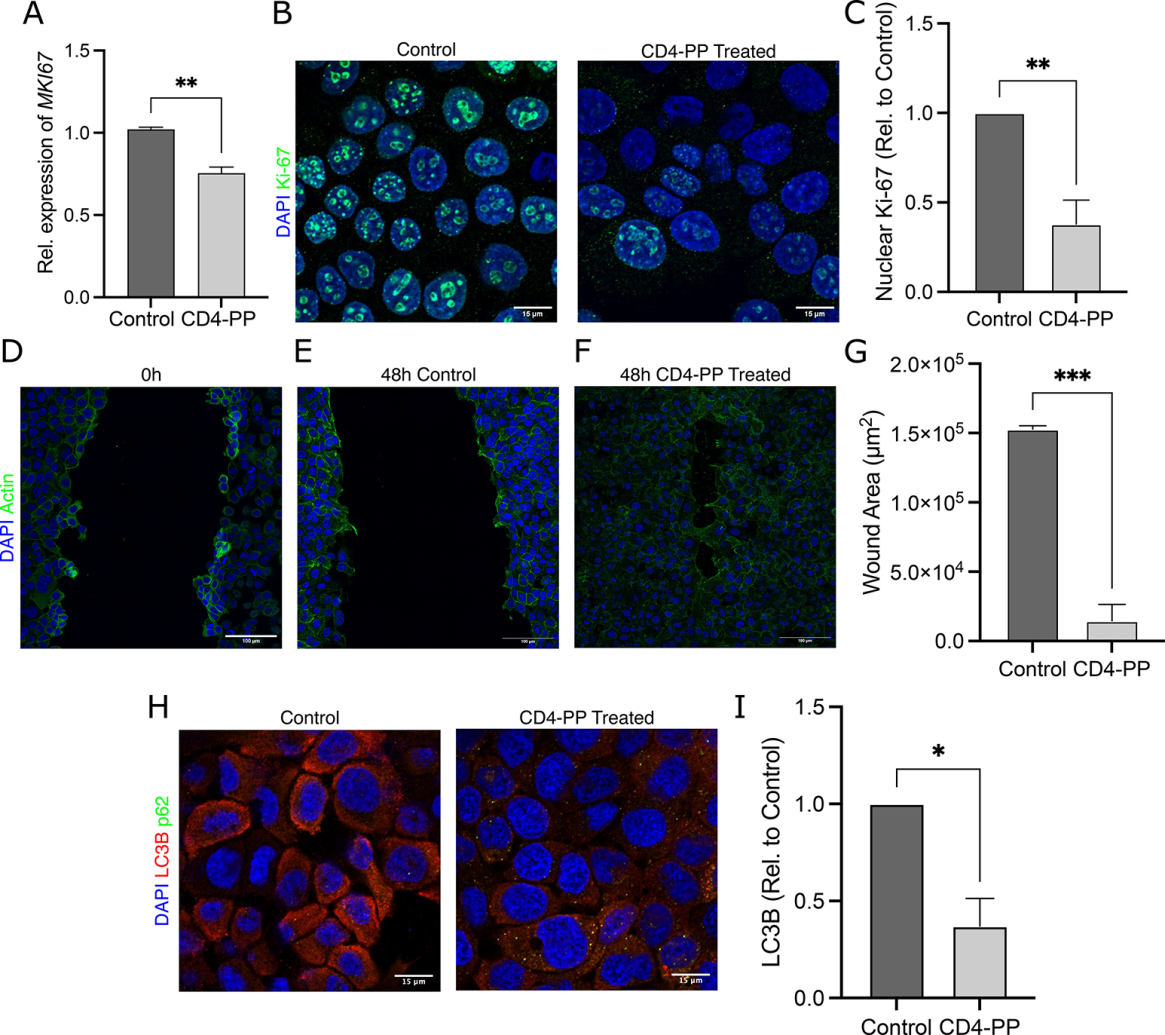
CD4-PP induces wound closure and clears *in vitro* infection. (A) Uninfected keratinocytes stimulated with CD4-PP showed a decrease in Ki-67 on the mRNA level. (B) Representative confocal imaging of Ki-67 protein expression shows a decrease in nuclear Ki-67 in CD4-PP-treated keratinocytes, depicting Ki-67 (green) and nucleus (blue). (C) Densitometric analysis of nuclear Ki-67 staining in keratinocytes with or without CD4-PP treatment. (D) Representative confocal images of keratinocytes fixed immediately postscarring. (E,F) Representative confocal images of MRSA-infected keratinocytes with or without CD4-PP treatment 48 h after scarring, depicting actin (green) and nucleus (blue). (G) Wound area size of MRSA-infected keratinocytes with or without CD4-PP treatment 48 h postscarring, showing significantly decreased wound area. (H) Representative confocal images of keratinocytes infected with MRSA CCUG 31966 with or without CD4-PP treatment, depicting nucleus (blue), LC3B (red), and p62 (green). (I) Densitometric analysis of LC3B in MRSA-infected keratinocytes with or without CD4-PP treatment. All microscopy imaging and densitometry analysis were performed in three independent experiments, with each experiment consisting of 4–5 random view fields. The average integrated density of each cell per set was used for statistical analyses (**p < 0.01, ***p < 0.001, unpaired *t* test).

To investigate the effect of CD4-PP on wound healing, a lawn of fully confluent keratinocytes was scarred and infected with MRSA. Treatment with CD4-PP significantly decreased the wound area compared with untreated controls (Figures 3D–G). In addition to keratinocytes, fibroblasts play an important role in wound repair through tissue remodelling and the secretion of wound healing factors, including VEGF and angiopoietin. Interestingly, we found that human dermal fibroblasts (hDF) infected with MRSA had no change in *VEGFA* expression and a significant decrease in *ANGPT1* expression (Supplementary Figure 1 E,F). However, MRSA-infected keratinocytes treated with CD4-PP showed an increase in both *VEGFA* and *ANGPT1* (Supplementary Figure 1G,H). This shows that while CD4-PP, itself, might decrease *K*i-67 in infected keratinocytes or angiopoietin in hDF, treatment of infected keratinocytes with CD4-PP was capable of increasing the expression of VEGF and angiopoietin in addition to reducing the wound area in infected keratinocytes. This observation is in line with other studies, which have shown that AMPs are capable of promoting wound closure.^19^

Autophagy is known to be involved in the maintenance of epithelial tissue by breaking down intracellular components to maintain homeostasis.^20^ During MRSA infection, we observed significantly more LC3B puncta in keratinocytes not treated with CD4-PP, while no difference was observed for p62, which is normally inversely proportional to LC3B expression (Figures 3H–I). The accumulation of LC3B is associated with the activation of autophagy, which has been suggested to inhibit cell growth through protein/organelle turnover.^21^ Therefore, a decrease in LC3B, as observed in CD4-PP-treated keratinocytes, might induce cell growth and lead to closure of the wound in infected keratinocytes.^14^

CD4-PP was designed as a backbone cyclic dimer peptide based on the shortest antimicrobial sequence of LL-37,^4,22^ called KR-12.^23^ Two KR-12 units were then connected using linkers of four amino acids into a seamless chain of peptide bonds. This design was inspired by naturally occurring antimicrobial cyclic peptides and their advantages of stability and, in some cases, increased cell permeability for drug development. The most well-known examples of such peptides are the cyclotides^24^ and the θ-defensins,^25^ which both have been subjects for peptide engineering. Recent examples include the grafting of porcine protegrin into the cyclotide scaffold to obtain broad spectrum AMPs with effect *in vivo*(26) and a minimized version of rhesus θ-defensin 1 (RTD-1) with effect against carbapenem-resistant Enterobacteriaceae sepsis.^27^ However, while these examples share a cyclic backbone, they represent different structural classes: RTD-1 and protegrin are both β-sheet peptides, while cyclotides may be described as more globular peptides, and they are all constrained by disulfide bonds. In contrast, the design of CD4-PP is based on two α-helical peptide monomers, whose structure is maintained when the peptide is in contact with lipid (cell) membranes.^4^

Overall, we demonstrate that CD4-PP has strong antibacterial activity against common skin pathogens, including those that are drug-resistant. The increased innate immune response seen in keratinocytes further contributes to clear bacterial infections caused by skin pathogens. Our results show that CD4-PP performs similarly with other examples of synthetic AMPs derived from endogenous peptides and shows both a direct effect on pathogens and modifies host-mediated inflammation.^28,29^ However, the versatility of CD4-PP against dermal and urinary pathogens, combined with the previously demonstrated stability,^4^ its effect on varying cell types, and its low cytotoxicity, is noteworthy. In addition, stimulation of infected keratinocytes with CD4-PP promotes cell proliferation in an *in vitro* scratch model. As an engineered AMP, CD4-PP displays potential for use in treating dermal infections and promoting wound closure.

## Methods

### Peptide Synthesis

The peptide CD4-PP was designed based on the shortest active region of LL-37, and synthesized using the methods as described previously.^30^

### Bacterial Strains and Cultures

The following bacterial skin pathogens were used: multidrug-resistant (MDR) *Acinetobacter baumannii*, *Staphylococcus aureus* (ATCC 29213), methicillin-resistant *Staphylococcus aureus* (MRSA; CCUG 31966), and *Streptococcus pyogenes* (group A streptococci; GAS; ATCC 19615). Clinical isolates were obtained from the Department of Clinical Microbiology, Karolinska University Hospital, Solna, Sweden. All clinical isolates were species identified using biochemical typing and MALDI-TOF MS. MRSA clinical strains were first identified as *S. aureus* by MALDI-TOF MS and confirmed to be MRSA by the eazyplex MRSA kit (Amplex Diagnostics, Germany) through detection of nuc and mecA or mecC cassettes and cefoxitin disk diffusion. *S. aureus*, and MRSA clinical isolates and type strains were cultured aerobically overnight on blood agar plates at 37 °C. All GAS isolates were grown on blood agar plates with gentamicin anaerobically at 37 °C.

### Minimum Inhibitory Concentration (MIC) Assay

The MIC of CD4-PP was evaluated against the type strains and the 20 clinical bacterial isolates of each species using a two-step microdilution assay adapted for testing AMPs using the methods as described previously.^4^

### Development of Immediate Resistors

To determine whether CD4-PP-sensitive isolates could quickly develop resistance, random clinical isolates of *S. aureus*, MRSA, and GAS were subcultured in TSB media supplemented with CD4-PP at MIC for 1 week, whereafter the MIC was repeated for these isolates using the methods as described previously.^31^

### Scanning Electron Microscopy

Scanning electron microscopy (SEM) was used to evaluate bacterial morphology after treatment with CD4-PP. SEM was performed on the basis of the methods described previously.^4^*S. aureus* ATCC 29213, GAS ATCC 19615, and MRSA CCUG 31966 were grown to mid-log phase and, thereafter, diluted in 10 mM Tris buffer at a cell density of 10^8^ cfu/mL. Bacterial suspensions (100 μL) were then incubated with CD4-PP (final concentration 3.9 μM for *S. aureus* and GAS and 7.8 μM for MRSA) for 1 h at 37 °C. The peptide concentration corresponded to the MIC found for the high cell density condition (10^8^ cfu/mL). Untreated bacteria served as the control and were used as reference. After the exposure experiment, bacterial suspensions (100–200 μL) were deposited on Nunc Thermanox coverslips (Thermofisher Scientific) and left to adhere for 1 h. Bacterial cells were then fixated with 4% paraformaldehyde (VWR Chemicals, USA) in PBS overnight at 4 °C, washed 2× with PBS and deionized water, postfixated with 1% osmium tetroxide (Sigma-Aldrich, USA) for 1 h, and washed with PBS and deionized water. The samples were then dehydrated with a series of ethanol concentrations (10, 30, 50, 70, 90, and 100% v/v), followed by further dehydration with hexamethyldisilazane (HMDS; Sigma-Aldrich) solutions (HMDS/ethanol = 1:2, 2:1, and 100% HMDS). HMDS solution was then removed, and the samples were left to air-dry overnight.

Coverslips were mounted on carbon stubs and sputter-coated with a conductive thin layer of gold and palladium. Bacterial cells were imaged using a LEO 1550 SEM instrument (Zeiss) with an InLens detector at 2–3 kV acceleration voltage and at 3–5 nm working distance.

### Estimation of Bacterial Metabolic Activity

The effect of 20 μM CD4-PP on the metabolic activity of *S. aureus* ATCC 29213 and MRSA CCUG 31966 was determined using an XTT assay. In brief, 50 μL from a bacterial suspension corresponding to a 0.5 McFarland standard was added to 150 μL of Tryptic soya broth (TSB) with a final concentration of 20 μM CD4-PP and kept at 37 °C for 24 h. Samples were then incubated with 200 μL of 20% solution of 1 mg/mL XTT (Sigma) in TSB for 4 h. The conversion of tetrazolium salt XTT to a colored formazan derivative was measured at 450 nm in a 96-well plate. Viability controls not treated with CD4-PP were maintained throughout the cell viability assay. Media blanks were subtracted from the test strains.

### Cell Culture

Human keratinocytes, HaCaT, (kindly provided by Prof. Mona Ståhle) were cultured in DMEM with 10% fetal bovine serum (FBS; Life Technologies). Human monocytes (THP1, ATCC TIB-202) were cultured in RPMI-1640 supplemented with 10% FBS. The THP1s were differentiated into macrophages (dTHP1) by stimulation with 150 ng/mL of phorbol 12-myristate 13-acetate (PMA) for 24 h. Human dermal fibroblasts (hDF) (purchased from Cell Applications, inc) were cultured in DMEM with 10% FBS. All cells were cultured at 37 °C with 5% CO_2_.

### RNA Extraction and Real-Time PCR Analysis

RNA extraction, cDNA synthesis, and quantitative PCR (qPCR) were performed as previously described.^4^ Gene targets used in this study included psoriasin (*S100A7*; fwd 5′-CAC CAG ACG TGA TGA CAA-3′, rev 5′-GGC TAT GTC TCC CAG CAA), IL8 (*CXCL8*; fwd 5′-GGC TAT GTC TCC CAG CAA-3′, rev 5′-GAT ATT CTC TTG GCC CTT GG-3′), Ki-67 (*MKI67*; fwd 5′-TCC CGC CTG TTT TCT TTC TGA C-3′, rev 5′-CTC TCC AAG GAT GAT GAT GCT TTA C-3′), VEGF (*VEGFA;* fwd 5′-CTT GTT CAG AGC GGA GAA AGC-3′, rev 5′-ACA TCT GCA AGT ACG TTC GTT-3′), and angiopoietin (*ANGPT1;* fwd 5′-GAC AGA TGT TGA GAC CCA GGT A-3′, rev 5′-TCT CTA GCT TGT AGG TGG ATA ATG AA-3′). Human beta actin (*ACTB*; fwd 5′-AAG AGA GGC ATC CTC ACC CT-3′, rev 5′-TAC ATC GCT GGG GTG TTG-3′) was used as the housekeeping gene to calculate relative gene expression.

### Immunofluorescence Microscopy

HaCaT cells were treated with CD4-PP for 24 h to assess Ki-67 expression. Cells were fixed with 4% paraformaldehyde, stained with anti-Ki-67 antibody (Abcam) at 1:200 dilution, followed by Alexa Fluor 488 secondary antibody at 1:1000 dilution, and counterstained with 4′,6-diamidino-2-phenylinodole (DAPI; Life Technologies). For psoriasin staining, HaCaT cells were infected with MRSA CCUG 31966 at multiplicity of infection (MOI) 10 with or without CD4-PP treatment before fixing. Cells were stained with anti-psoriasin (Santa Cruz) at 1:200 dilution, followed by Alexa Fluor 488 secondary antibody at 1:400 dilution, and counterstained with DAPI. For LC3B and p62 staining, HaCaT cells were treated with or without CD4-PP for 24 h overnight before infection with MRSA CCUG 31966 at MOI 5 for 2 h. Cells were stained with anti-LC3B (Novus Biologicals) and anti-p62 (Santa Cruz) at 1:200 dilution, followed by Alex Fluor 594 and 647 secondary antibodies at 1:400 dilution, and counterstained with DAPI. Confocal imaging was performed using the 63× oil immersion objective of a Zeiss LSM 700 microscope (Carl Zeiss). The fluorescence intensity and area of each cell were quantified by manually defining the boundaries around each cell with the FIJI software.^32^ For each cell, the intensity/area was calculated. Since each view field contained 5–10 cells, the average intensity/area of all cells in the view field was calculated. The expression of the target value was then normalized to a corresponding control average value, thereby making the control as 1. At least three or four view fields per slide were quantified from three independent experiments.

### *In Vitro* Wound Closure Model

For the assessment of wound closure *in vitro*, a straight line scratch was made on a lawn of fully confluent HaCaT cells. The wound was infected with MRSA CCUG 31966 at MOI 1 for 6 h before the removal of unattached bacteria via washing with PBS. Cells were further incubated for 48 h in media with or without 10 μM CD4-PP. The media was replaced every 24 h until cell fixation. Cells were stained with DAPI and Alexa Fluor 488 phalloidin (1:1000). Imaging was performed with the 63× oil immersion in the 5 × 5 tile mode of an LSM 700 microscope. The wound area was determined using the Wound Healing Size Tool plugin in Fiji.^33^

### Cell Infection Assays

HaCaT, hDF, and dTHP1 cells were seeded at ∼80% confluency in 24-well plates (Costar). Cells were infected with the aforementioned bacterial type strains at MOI 5 using the methods as described previously.^4^

### Statistical Analysis

All statistical tests were performed in GraphPad Prism, version 9.4.1. Statistical outliers, as defined by Grubb’s test, were excluded from the data sets. Statistical comparisons between two variables were performed by paired *t* test where appropriate, whereas those involving multiple comparisons were done by one-way ANOVA. Differences with a *p*-values less than 0.05 were regarded as being statistically significant.

## Supporting Information Available

Representative image of keratinocytes infected with *S. aureus* depicting psorasin peptide released from keratinocytes colocalizing with *S. aureus*, bacterial survival in macrophages treated with CD4-PP, and VEGF and angiopoietin expression in MRSA infected keratinocytes and dermal fibroblasts (PDF)
