# Supplementary material for: A Synthetic Cyclized Antimicrobial Peptide with Potent Effects against Drug-Resistant Skin Pathogens
Source: ACS Infect Dis. 2023 May 3;9(5):1056–63. doi: 10.1021/acsinfecdis.2c00598 (PMC10186370; doi:10.1021/acsinfecdis.2c00598)
Supplement: Supplementary file 1 — id2c00598_si_001.pdf [file id2c00598_si_001.pdf]

Supporting Information

A synthetic cyclized antimicrobial peptide with potent effects against drug resistant skin pathogens

John Kerr White[1,2], Soumitra Mohanty[1,2,3], Taj Muhammad [3,4], Magdalena de Arriba Sanchez de la Campa[1,2], Wael E. Houssen[4,5], Natalia Ferraz[6], Ulf Göransson[3], Annelie Brauner[1,2*]

[1] Department of Microbiology, Tumor and Cell Biology, Karolinska Institutet, SE-171 77 Stockholm, Sweden.

[2] Division of Clinical Microbiology, Karolinska University Hospital, SE-171 76 Stockholm, Sweden.

[3] Pharmacognosy, Department of Pharmaceutical Biosciences, Uppsala University, Biomedical Centre, Box 591 SE-75124 Uppsala, Sweden

[4] Institute of Medical Sciences, University of Aberdeen, Ashgrove Road West, Foresterhill Aberdeen AB25 2ZD, UK

[5] Department of Chemistry, University of Aberdeen, Meston Walk, Aberdeen AB24 3UE, UK.

[6] Nanotechnology and Functional Materials, Department of Materials Science and Engineering, Uppsala University, Box 35, SE-75103 Uppsala, Sweden

* Corresponding author

Annelie Brauner, Department of Microbiology, Tumour and Cell Biology, Division of Clinical Microbiology, Karolinska Institutet and Karolinska University Hospital, 17176 Stockholm, Sweden; Phone +46 8 51770000, Fax: +46 8 308099 E-mail: Annelie.Brauner@ki.se

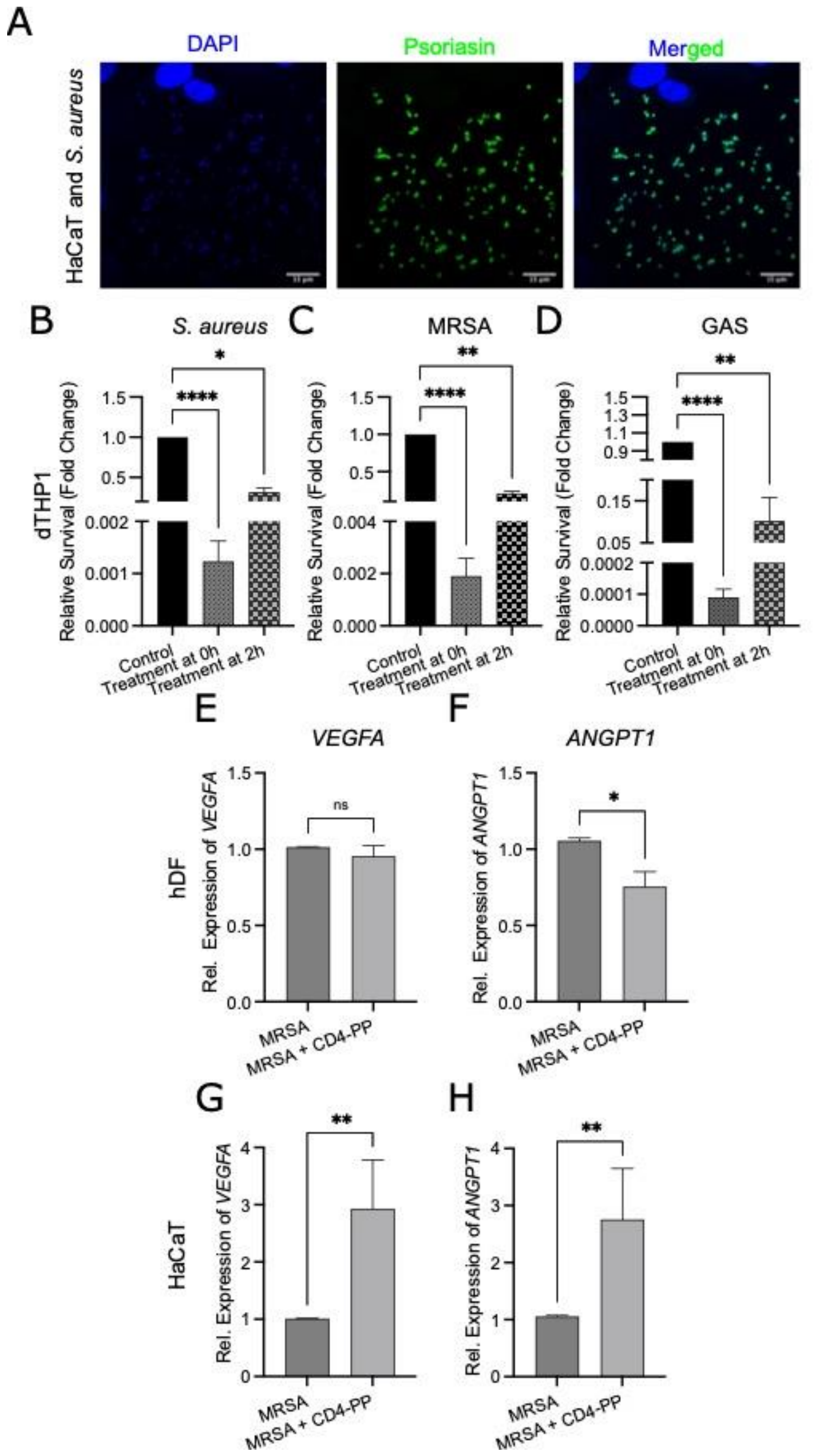

Figure S1

Representative image of keratinocytes infected with S. aureus depicting psorasin peptide released from keratinocytes co-localising with S. aureus. Survival of skin pathogens (B) S. aureus (ATCC 29213), (C) MRSA (CCUG 31966), and (D) GAS (ATCC 19615) after infecting macrophages (dTHP1s).

Survival in treatment groups is relative to untreated control. CD4-PP was initiated at the same time as infection (Treatment at 0 h) or after 2 h of infection (Treatment after 2 h). Influence of CD4-PP on the gene expression of VEGFA and ANGPT1 in human dermal fibroblasts (hDF) and keratinocytes (HaCaT), infected with MRSA (CCUG 31966, E-H) $p<0.05$, paired t-test.
